# Intraspecific identification of *Actinidia eriantha* Benth. based on chloroplast genes

**DOI:** 10.1371/journal.pone.0342803

**Published:** 2026-02-23

**Authors:** Lan Lan, Jiale Mao, Bingyan Mao, Shuang Liu, Tao Li, Xiaohui Zhou, Houxing Lei, Songquan Wu, Jia Chen, Xiaoqin Zhang

**Affiliations:** 1 Department of Pharmacy, Lishui TCM Hospital Affiliated to Zhejiang Chinese Medical University (Lishui Hospital of Traditional Chinese Medicine), Lishui, Zhejiang, China; 2 School of Life Sciences, Zhejiang Chinese Medical University, Hangzhou, Zhejiang, China; 3 School of Medicine, Lishui University, Lishui, Zhejiang, China; Institute for Biological Research, University of Belgrade, SERBIA

## Abstract

**Objective:**

This study aimed to identify specific DNA barcodes based on the chloroplast genome of *Actinidia eriantha* Benth. and to utilize these barcodes for the identification of germplasm resources from different geographic origins.

**Methods:**

The chloroplast genome of *A. eriantha* samples were sequenced using the Illumina NovaSeq PE150 platform. Specific highly variable regions were identified through mVISTA alignment and nucleotide diversity analysis. Haplotypes of samples from various regions were further analyzed based on the selected DNA barcode candidate fragments.

**Results:**

The complete chloroplast genomes of three *A. eriantha* from different locations were 156,955–157,100 bp in length and exhibited a typical quadripartite circular structure, with 88 genes annotated in each genome. Comparative analyses with mVISTA and nucleotide diversity indices identified *matK*, *trnK(UUU)*, *ycf1*, and the *atpH_atpI* intergenic spacer as candidate regions for specific DNA barcodes. Among these, *trnK(UUU)*, *ycf1*, and *atpH_atpI* were selected for further analysis based on PCR amplification efficiency. Sequencing of these three regions across 223 samples from 21 locations in six provinces revealed 7, 10, and 39 polymorphic sites, respectively, which defined 6, 4, and 6 haplotypes. A combined analysis of the three loci identified 56 polymorphic sites and 12 distinct haplotypes (Hap1-Hap12), with pairwise genetic distances ranging from 0 to 1.96%. Six haplotypes were found to be unique to specific geographic regions, suggesting their potential as molecular markers for tracing the geographic origin of *A. eriantha*.

**Conclusion:**

The chloroplast gene regions *trnK(UUU)*, *ycf1*, and *atpH_atpI*, identified through comparative chloroplast genomics, serve as effective DNA barcodes for the intraspecific identification of *A. eriantha* germplasm. These markers provide a molecular basis for future efforts in geographic origin tracing, germplasm conservation, and breeding of this species.

## Introduction

The root of *Actinidia eriantha* Benth., commonly known as “*Baishan Maotao Root*,” is a traditional medicinal herb used by the She ethnic minority in China [1] and is officially listed in the 2015 edition of the *Zhejiang Provincial Standards for the Processing of Traditional Chinese Medicine*. Pharmacologically, it is traditionally employed to clear heat and detoxify, promote urination and reduce swelling, and is used in the ethnomedicine of the She people to treat mastitis, ascites, rheumatic arthralgia, and traumatic injuries.

Modern pharmacological studies have demonstrated that *A. eriantha* exhibits a wide range of biological activities, including anti-tumor, anti-inflammatory, antioxidant, anti-angiogenic, immunomodulatory, and neuroprotective effects [2]. Notably, it has shown therapeutic potential in the treatment of various cancers such as gastric cancer [3,4], colorectal cancer [5], breast cancer, nasopharyngeal carcinoma [6], and leukemia [2], highlighting its promise as a candidate for anticancer drug development. In recent years, clinical demand for *A. eriantha* has increased substantially. However, due to the lack of cultivation bases, wild populations have declined sharply. Genetic diversity studies based on internal transcribed spacer (ITS) sequences have indicated that *A. eriantha* exhibits relatively low genetic diversity [7], underscoring the urgent need for genetic conservation strategies. A crucial step in the conservation and sustainable utilization of *A. eriantha* genetic resources is the accurate intraspecific identification of germplasm from different geographical origins. This is essential for resource authentication, cultivar selection, and breeding. Traditional methods for intraspecific identification include morphological identification (e.g., macroscopic and microscopic examination) and chemical profiling (e.g., chromatographic or spectroscopic techniques). However, morphological methods are highly susceptible to environmental conditions and developmental stages, often resulting in low efficiency and high subjectivity, particularly when distinguishing among closely related taxa. Chemical methods, while more precise, are limited by complex sample preparation, high costs, poor compound stability, and limited discriminatory power for closely related species.

Comparative chloroplast genomics based on the combination of chloroplast genome sequencing and DNA barcoding has emerged as an effective strategy for screening high-resolution DNA barcodes [8]. Chloroplasts are plant cell organelles that possess their own genetic material, independent of the nuclear genome. Compared with nuclear DNA, chloroplast genomes are structurally more stable, relatively small in molecular weight, and exhibit conserved gene content and organization. In most angiosperms, the chloroplast genome is composed of highly conserved double-stranded circular DNA that exhibits a typical quadripartite structure, consisting of a large single-copy (LSC) region, a small single-copy (SSC) region, and a pair of inverted repeat regions (IRa and IRb) [9–11]. Due to their overall structural conservation combined with localized sequence variability, chloroplast genomes are widely recognized as powerful tools for studying plant genetic diversity and reconstructing phylogenetic relationships among taxa [12]. By systematically comparing the complete chloroplast genomes of the same species from different geographic origins, researchers can identify hypervariable regions with significant sequence variations, thereby screening for specific barcodes suitable for species authentication. This approach has been successfully applied to geographic traceability and authenticity identification in various medicinal plants and crops. For instance, Wang et al. compared the chloroplast genomes of *Fritillaria ussuriensis* from different regions and identified the highly variable *atpF* and *petB* genes as specific barcodes, effectively distinguishing samples from different origins and providing molecular evidence for the geographical authenticity of the medicinal material [13]. An et al. [14] employed comparative chloroplast genomics to obtain two specific DNA barcodes, *rpoB* and *psbK-psbI*, which exhibited the highest mutation rates in *Paeonia lactiflora*. Combined analysis of these two fragments revealed 15 haplotypes in wild populations, enabling effective differentiation of *P. lactiflora* from different origins. He et al. [15], based on the chloroplast genes *atpF* and *rps4-trnT-UGU* for intraspecific identification of *Salvia miltiorrhiza* from different regions, discovered 19 haplotypes unique to specific production areas. These region-specific haplotypes can serve as DNA barcode markers for regional identification.

Notably, the chloroplast genome typically exhibits maternal inheritance in most angiosperms, and is consequently widely employed for reconstructing maternal lineage history and seed dispersal patterns. However, in certain taxa such as the *genus Actinidia*, chloroplasts display the less common paternal or biparental inheritance. The study species *A. eriantha* has been preliminarily identified as possessing paternally inherited chloroplasts. This unique inheritance pattern offers a valuable opportunity to directly investigate pollen-mediated paternal gene flow and phylogeographic structure using chloroplast DNA sequences—questions that traditionally require indirect inference through nuclear genomic or biparentally inherited markers.

To date, several chloroplast genome sequences have been reported within the genus *Actinidia*, including *A. eriantha* [12], *A. latifolia* (Gardner & Champ.) Merr. [16], *A. styracifolia* C. F. Liang [17], and *A. rubus* Levl. [18]. However, studies on specific DNA barcodes based on comparative chloroplast genomics of *A. eriantha* have not been reported. Therefore, this study selected three *A. eriantha* samples from different geographical origins for chloroplast genome sequencing. Comparative genomic analysis was performed to identify specific DNA barcodes, which were subsequently applied to germplasm identification of 223 *A. eriantha* samples collected from 21 locations across six provinces. This work establishes a groundwork for future germplasm screening, quality control, and related research on *A. eriantha.*

## Materials and methods

### Plant materials

**Fig 1:**
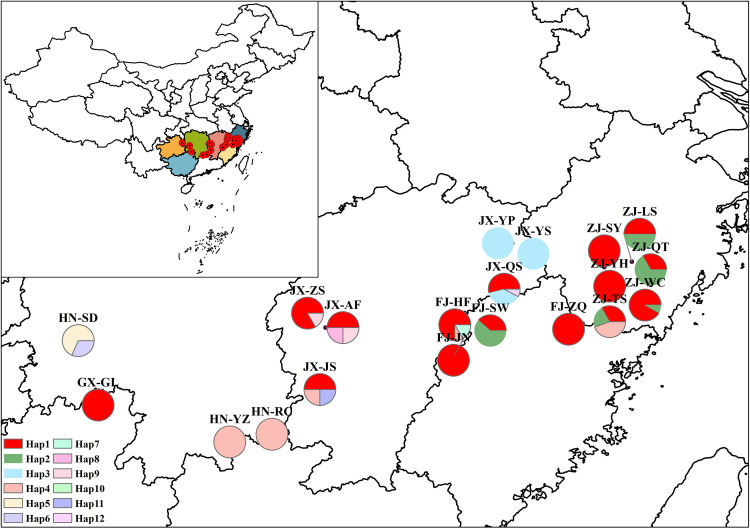
Sampling locations and distribution frequencies of haplotypes reconstructed from the combined analysis of three chloroplast DNA regions (*trnK(UUU)*, *ycf1*, and *atpH_atpI*) in *A. eriantha.* Population details correspond to those in Table 2. This map is based on the standard map with the review number GS(2020)4619 downloaded from the Standard Map Service website of the National Bureau of Surveying and Mapping Geographic Information (http://bzdt.ch.mnr.gov.cn/). The base map has not been modified.

Three *A. eriantha* samples were collected from Huaihua City in Hunan Province, Ji’an City in Jiangxi Province, and Lishui City in Zhejiang Province for complete chloroplast genome sequencing (Table 1). A total of 223 samples from 21 geographic locations were used for germplasm identification, with their origins and haplotype distributions summarized in Table 2 and Fig 1. All plant materials were taxonomically identified as *A. eriantha* by Chief Pharmacist Lin Na from Lishui Municipal Hospital of Traditional Chinese Medicine. No specific permits were required for the collection of the plant leaf samples in this study.

**Table 1:** Source of chloroplast genome sequencing samples from *A. eriantha.*

Name	Locatiy information	Longitude	Latitude	Alt. (m)
*A. eriantha* 1	Sobaoding, Huaihua, Hunan	110°20’E	27°8’N	1279.6
*A. eriantha* 2	Anfu, Jian, Jiangxi	114°36’E	27°35’N	315.7
*A. eriantha* 3	Lishan, Liandu, Lishui, Zhejiang	119°57’E	28°32’N	338.2

**Table 2:** Sampling information of *A. eriantha* samples and haplotype distribution based on the combined analysis of three chloroplast DNA regions (*trnK(UUU)*, *ycf1*, and *atpH_atpI*).

Locatiy information	Samples No.	Samples size	Haplotype(number)
Rucheng, Chenzhou, Hunan	RC	4	Hap4(4)
Yizhang, Chenzhou, Hunan	YZ	3	Hap4(3)
Subaoding, Huaihua, Hunan	SD	19	Hap5(13), Hap6(6)
Anfu, Jian, Jiangxi	AF	4	Hap1(2), Hap8(1), Hap9(1)
Suichuan, Jian, Jiangxi	JS	4	Hap1(2), Hap4(1), Hap11(1)
Qianshan, Shangrao, Jiangxi	QS	13	Hap1(7), Hap3(5), Hap12(1)
Yangjiaping, Dexing, Jiangxi	YP	3	Hap3(3)
Yushan, Shangrao, Jiangxi	YS	6	Hap3(6)
Laozhushan, Yichun, Jiangxi	ZS	6	Hap1(5), Hap9(1)
Ziyuan, Guilin,Guangxi	GX	3	Hap1(3)
Jiangkou, Tongren, Guizhou	GZ	58	Hap1(49), Hap2(8), Hap10(1)
Huangfang, Jianning, Sanming, Fujian	HF	12	Hap1(9), Hap4(1), Hap7(2)
Suixi, Jianning, Sanming, Fujian	JN	15	Hap1(15)
Zhenghe, Nanping, Fujian	ZQ	10	Hap1(10)
Shaowu, Nanping, Fujian	SW	13	Hap1(5), Hap2(8)
Lishan, Liandu, Lishui, Zhejiang	LS	2	Hap1(1), Hap2(1)
Qingtian, Lishui, Zhejiang	QT	9	Hap1(3), Hap2(6)
Songyang, Lishui, Zhejiang	SY	5	Hap1(5)
Taishun, Wenzhou, Zhejiang	TS	9	Hap1(3), Hap2(2), Hap4(4)
Wencheng, Wenzhou, Zhejiang	WC	12	Hap1(11), Hap2(1)
Yunhe, Lishui, Zhejiang	YH	13	Hap1(13)

### Extraction of chloroplast genomic DNA

Total genomic DNA was extracted from fresh leaves using the SDS method. DNA quality and concentration were assessed using agarose gel electrophoresis and a NanoDrop One spectrophotometer (Thermo Fisher Scientific).

### Bioinformatic isolation, assembly, sequencing, and annotation of the chloroplast genome

The chloroplast genome was de novo assembled from the raw reads generated by the whole-genome sequencing described above using a bioinformatic approach. The DNA samples that passed quality control via electrophoretic analysis were randomly sheared into fragments of approximately 350 bp using a Covaris ultrasonicator. Fragmented DNA was subjected to end-repair, A-tailing, adaptor ligation, purification, and PCR amplification using the NEBNext^®^ Ultra^TM^ DNA Library Prep Kit for Illumina (Cat# 7103, NEB). The prepared libraries were sequenced on an Illumina NovaSeq platform with a paired-end 150 bp (PE150) read strategy. Raw sequencing reads were processed through a four-step quality control pipeline: (1) removal of adapter sequences, (2) trimming of bases with a quality score ≤20, (3) discarding reads shorter than 50 bp or lacking paired ends, and (4) retention of clean reads for downstream analysis. Clean reads were assembled into chloroplast genomes using the GetOrganellev1.7.7.1 (https://github.com/Kinggerm/GetOrganelle) [19] with a maximum extension round of 15. Default parameters were applied for the K-mer size and reference database. Assembly was performed under a Linux environment, and the best assembly based on K-mer performance was selected for annotation. Annotation of the assembled chloroplast genomes was performed using the Plastid Genome Annotator (PGA). Sequence reads were mapped against the reference chloroplast genome of *A. eriantha* (GenBank Accession: NC_046774) using the Burrows-Wheeler Aligner (BWA). A circular map of the complete chloroplast genome was generated using the Organellar Genome DRAW (OGDRAW) tool.

### Whole chloroplast genome comparative analysis

Whole-genome alignment of the three *A. eriantha* chloroplast genome sequences was conducted using the mVISTA program, with one sequence as the reference. To evaluate nucleotide variability among the genomes, sliding window analysis was performed using DnaSP v6. The nucleotide diversity (*Pi*) values were calculated across the aligned chloroplast genomes to identify highly variable regions.

### Amplification of hypervariable regions

The hypervariable regions identified from the chloroplast genome comparison were used as molecular markers to assess the genetic differentiation of *A. eriantha* germplasm from different geographic regions. Specific primers (Table 3) were designed based on these variable loci, and PCR amplification was conducted for all 223 samples collected from 21 regions. PCR reactions were performed in a 50 µL volume containing: 35.5 µL ddH_2_O, 5 µL 10 × buffer, 4 µL dNTPs, 2 µL forward primer, 2 µL reverse primer, 1 µL genomic DNA template, and 0.5 µL TaKaRa Taq polymerase. The thermal cycling protocol was as follows: initial denaturation at 95°C for 3 min; followed by 30 cycles of 98°C for 10 s, 55°C for 30 s, and 70°C for 1 min; with a final extension at 72°C for 5 min. PCR products were stored at 4°C prior to further processing. Amplicons were purified and sequenced by Sangon Biotech Co., Ltd. (Shanghai, China).

**Table 3:** Primer information of three genes of *A. eriantha.*

Gene name	Primer sequences(5’–3’)	Annealing temperature (°C)
*trnK(UUU)*	Forward: ACTATTTCTTTCCCTTCTAG	55
	Reverse: AGGATTTCTAACCGTCTT	
*ycf1*	Forward: GAAGGACAAGCACGAAGA	
	Reverse: AGCACGGAATGCGGAGTT	
*atpH_atpI*	Forward: ATTAAATTCCATAGACCTAG	
	Reverse: AAAGATGACGATTACGAG	

### Phylogenetic analyses

The resulting sequence chromatograms were manually checked and assembled using Chromas and DNAMAN software. A maximum likelihood (ML) phylogenetic tree was constructed using MEGA X software. The built-in model selection function in MEGA X was employed to compare the fit of 24 different nucleotide substitution models. Based on the calculated Bayesian Information Criterion (BIC) and corrected Akaike Information Criterion (AICc), the model with the lowest BIC score was selected as the optimal model. The analysis indicated that the Tamura 3-parameter (T92) model best described the substitution pattern of the data. Bootstrap analysis was performed with 1,000 replicates, and the tree topology was automatically computed by the software.

## Results

### General features of the *A. eriantha* chloroplast genome

**Fig 2:**
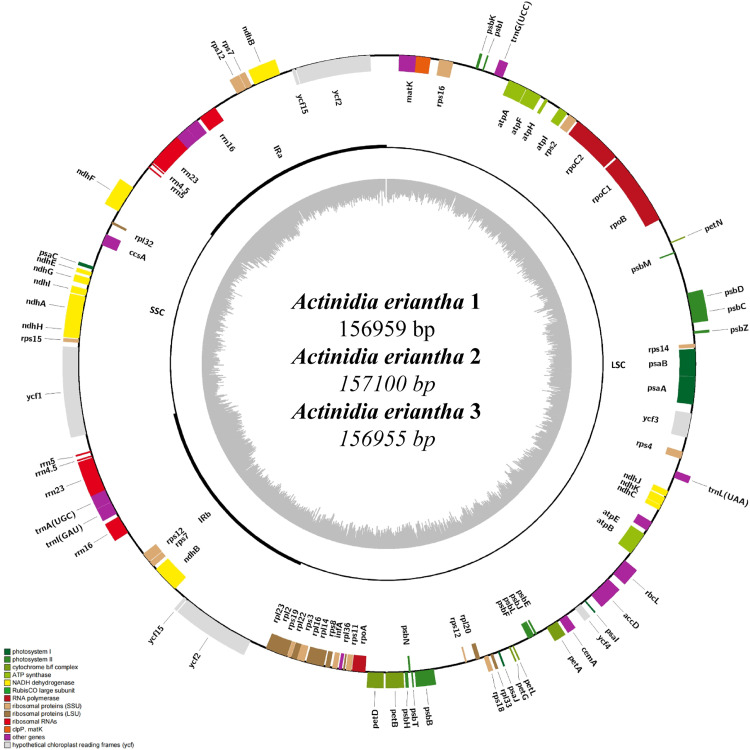
Circular maps of the concatenated complete chloroplast genomes in three *A. eriantha* samples. From the innermost to the outermost circle: the first ring represents GC content; the second ring (inner) shows genes, rRNAs, and tRNAs on the reverse strand; the second ring (outer) shows annotations on the forward strand. Colored blocks denote different functional categories. LSC: large single-copy region; SSC: small single-copy region; IRa/IRb: inverted repeat regions.

After quality filtering and adapter trimming, the raw sequencing data from *A. eriantha* Benth. samples *A. eriantha* 1 (HN), *A. eriantha* 2 (JX), and *A. eriantha* 3 (ZJ) yielded 104,073,964 (15.61 GB), 92,267,420 (13.84 GB), and 65,621,944 (9.84 GB) clean reads, respectively. Assembly of the chloroplast genomes from the three *A. eriantha* samples resulted in typical circular quadripartite structures. The total lengths of the complete chloroplast genomes were 156,959 bp (*A. eriantha* 1), 157,100 bp (*A. eriantha* 2), and 156,955 bp (*A. eriantha* 3), respectively, as illustrated in Fig 2. Each genome displayed the canonical quadripartite architecture, consisting of a large single-copy (LSC) region, a small single-copy (SSC) region, and two inverted repeat regions (IRa and IRb). The lengths of the LSC regions were 88,639 bp, 88,645 bp, and 89,478 bp, respectively; the SSC regions were 21,598 bp, 21,599 bp, and 20,537 bp; the IRa regions were 23,411 bp, 23,477 bp, and 23,137 bp; and the IRb regions were 23,312 bp, 23,379 bp, and 23,057 bp, respectively (Fig 2). The overall GC content of the chloroplast genomes for *A. eriantha* 1 and *A. eriantha* 3 was identical at 37.19%. The GC contents of the LSC and SSC regions of the chloroplast genome of *A. eriantha* 1 and *A. eriantha* 2 were identical, at 35.45% and 31.28, respectively. For *A. eriantha* 1, the GC content of the IRa and IRb regions was 43.28%. In *A. eriantha* 2, the total GC content was 37.17%, with IRa and IRb regions exhibiting GC contents of 43.20% each. The *A. eriantha* 3 chloroplast genome had GC contents of 35.43% (LSC), 31.09% (SSC), and 43.37% (IRa and IRb) (Table 4).

**Table 4:** Summary of chloroplast genome characteristics in three *A. eriantha* samples.

Samples	Genome		LSC		SSC		IRa		IRb	
	Length/bp	G + C/%	Length/bp	G + C/%	Length/bp	G + C/%	Length/bp	G + C/%	Length/bp	G + C/%
*A. eriantha* 1	156959	37.19	88639	35.45	21598	31.28	23411	43.28	23312	43.28
*A. eriantha* 2	157100	37.17	88645	35.45	21599	31.28	23477	43.20	23379	43.20
*A. eriantha* 3	156955	37.19	89478	35.43	20537	31.09	23137	43.37	23057	43.37

The three *A. eriantha* samples (HN, JX, and ZJ) showed average chloroplast genome sequencing depths of 4278.75, 3277.66, and 2708.15, respectively, with 88 genes annotated per assembly (Table 5), comprising 78 protein-coding genes, 6 transfer RNA (tRNA) genes, and 4 ribosomal RNA (rRNA) genes. The LSC region contained 62 protein-coding genes and four tRNAs, specifically *trnG-UCC*, *trnK-UUU*, *trnL-UAA*, and *trnV-UAC*. The SSC region harbored 11 protein-coding genes, including *ccsA*, *ndhA*, *ndhE*, *ndhF*, *ndhG*, *ndhH*, *ndhI*, *psaC*, *rpl32*, *rps15*, and *ycf1.* A total of 11 genes were duplicated in the inverted repeat (IR) regions, encompassing five protein-coding genes (*ndhB*, *rps12*, *rps7*, *ycf15*, and *ycf2*), two tRNA genes (*trnA-UGC* and *trnI-GAU*), and four rRNA genes (*rrn16*, *rrn23*, *rrn4.5*, and *rrn5*). Furthermore, 15 genes were identified to contain a single intron, including: *petD*, *rpl2*, *petB*, *ycf2*, *trnV-UAC*, *rpoC1*, *rpl16*, *trnG-UCC*, *trnK-UUU*, *trnL-UAA*, *atpF*, *rps16*, *ndhA*, *rps7*, and *ycf15*. Only one gene, *ycf3*, was found to contain two introns.

**Table 5:** Gene composition of the chloroplast genomes in three *A. eriantha* samples.

Category	Gene group	Gene name
Photosynthesis	Subunits of photosystem I	*psaA*, *psaB*, *psaC*, *psaI*, *psaJ*
	Subunits of photosystem II	*psbB*, *psbC*, *psbD*, *psbE*, *psbF*, *psbH*, *psbI*, *psbJ*, *psbK*, *psbL*, *psbM*, *psbN*, *psbT*, *psbZ*
	Subunits of NADH dehydrogenase	ndhA*, *ndhB* (2), *ndhC*, *ndhE*, *ndhF*, *ndhG*, *ndhH*, *ndhI*, *ndhJ*, *ndhK*
	Subunits of cytochrome b/f complex	*petA*, petB*, petD*, *petG*, *petL*, *petN*
	Subunits of ATP synthase	*atpA*, *atpB*, *atpE*, atpF*, *atpH*, *atpI*
	Large subunit of rubisco	*rbcL*
Self-replication	Proteins of large ribosomal subunit	*rpl14*, *rpl20*, *rpl22*, *rpl23*, *rpl32*, *rpl33*, *rpl36*, rpl16*, rpl2*
	Proteins of small ribosomal subunit	*rps11*, *rps12* (2), *rps14*, *rps15*, rps16*, *rps18*, *rps19*, *rps2*, *rps3*, *rps4*, rps7* (2), *rps8*
	Subunits of RNA polymerase	*rpoA*, *rpoB*, rpoC1*, *rpoC2*
	Ribosomal RNAs	*rrn16* (2), *rrn23* (2), *rrn4.5* (2), *rrn5* (2)
	Transfer RNAs	trnG-UCC*, trnK-UUU*, trnL-UAA*, trnV-UAC*, *trnA-UGC* (2), *trnI-GAU* (2)
Other genes	Maturase	*matK*
	Envelope membrane protein	*cemA*
	Acetyl-CoA carboxylase	*accD*
	c-type cytochrome synthesis gene	*ccsA*
	Translation initiation factor	*infA*
Genes of unknown function	Conserved hypothetical	*ycf1*, ycf2* (2), *ycf3*, *ycf4*

*: A gene containing one intron, * *: A gene containing two introns, (2): A gene with two copies.

### Comparative genomic analysis

**Fig 3:**
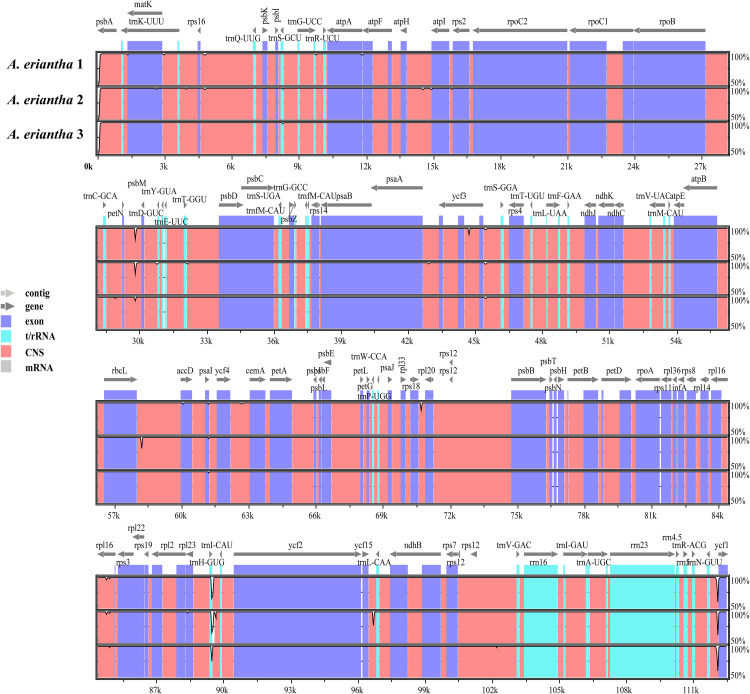
Sequence homology analysis of the chloroplast genomes in three *A. eriantha* samples.

**Fig 4:**
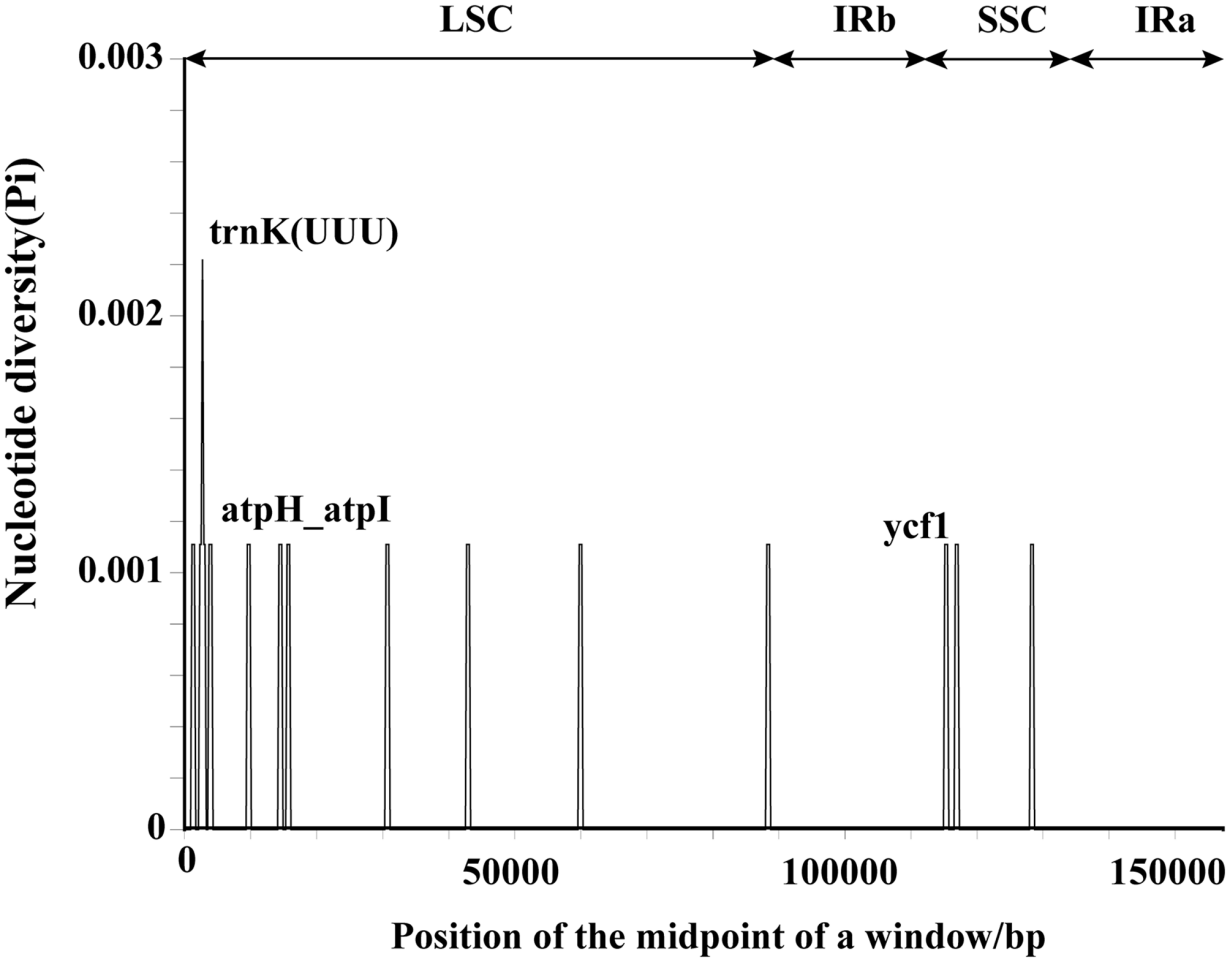
Nucleotide diversity of the chloroplast genomes in three *A. eriantha* samples.

To assess intraspecific variation among the three *A. eriantha* chloroplast genomes, a global alignment was performed using the mVISTA online platform, with the annotated *A. eriantha* chloroplast genome (GenBank accession: NC_034914) as the reference (Fig 3). The alignment revealed that coding regions were more conserved than non-coding regions, and sequence variation in the inverted repeat (IR) regions was significantly lower than that in the LSC and SSC regions. Most genes exhibited sequence similarity above 90%, and the rRNA genes (*rrn4.5*, *rrn5*, *rrn16*, and *rrn23*) were highly conserved, with no observed variation. However, variation was detected in several gene regions, including *matK*, *trnK(UUU)*, *ycf1*, *accD*, and *rpl23*, as well as in intergenic spacers such as *trnK(UUU)_rps16*, *trnG(UCC)_atpA*, *atpH_atpI*, *atpI_rps2*, *psbM_psbD*, *psaA_ycf3*, and *rrn5_rps15*. To further evaluate sequence divergence, *Pi* across the three genomes was calculated using DnaSP. The results indicated relatively low intraspecific variation, with *Pi* values ranging from 0.00000 to 0.00222. The average *Pi* values in the LSC, SSC, and IR regions were 0.00008, 0.00010, and 0, respectively, consistent with the patterns observed in the mVISTA analysis (Fig 4). Among all loci, the gene regions *trnK(UUU)* (*Pi* = 0.00222) and *ycf1* (*Pi* = 0.00111), as well as the intergenic region *atpH_atpI* (*Pi* = 0.00111), showed relatively higher nucleotide diversity. While the chloroplast genomes of *A. eriantha* show the expected high degree of intraspecific conservation due to the lack of recombination, the limited variations identified in regions such as *matK*, *trnK(UUU)* and *ycf1* are nonetheless informative for distinguishing haplogroups and inferring population structure. Non-coding regions generally exhibited higher sequence variation than coding regions. Based on both mVISTA and *Pi* analyses, the following loci were identified as candidate hypervariable regions for molecular marker development: coding regions *matK*, *trnK(UUU)*, *ycf1*, *accD*, and *rpl23*, and intergenic regions *trnK(UUU)_rps16*, *trnG(UCC)_atpA*, *atpH_atpI*, *atpI_rps2*, *psbM_psbD*, *psaA_ycf3*, and *rrn5_rps15*.

### DNA barcode analysis of samples from different geographical origins

Based on the candidate regions *matK* (1253 bp), *trnK(UUU)* (2549 bp and 2885 bp), *atpH_atpI* (14,433 bp and 14,839 bp), and *ycf1* (115,170 bp and 116,898 bp), specific primers were designed for PCR amplification. Among them, *matK* (1253 bp), *atpH_atpI* (14,839 bp), and *ycf1* (116,898 bp) exhibited low amplification efficiency. Therefore, the *trnK(UUU)* (2549 bp and 2885 bp), *ycf1* (115,170 bp), and *atpH_atpI* (14,433 bp) loci were selected as specific DNA barcodes for intraspecific identification of *A. eriantha* and were used in subsequent analyses. Using the designed primers, total genomic DNA was extracted from 223 *A. eriantha* samples for PCR amplification targeting the three selected barcode regions.

Analysis of the *trnK(UUU)* region revealed 7 polymorphic sites, all of which were single nucleotide polymorphisms (SNPs), located at positions 91, 98, 109, 155, 427, 435, and 438 bp. These variations resulted in the formation of 6 haplotypes, designated THap1 to THap6 (Table 6). For the *ycf1* gene, 10 polymorphic sites were identified, forming 4 haplotypes (YHap1-YHap4, Table 7). Among them, six contiguous sites (positions 274–279 bp) were deletions, while the remaining four sites at positions 139, 165, 171, and 264 bp were SNPs. The *atpH_atpI* region exhibited the highest variability, with 39 polymorphic sites, resulting in 6 haplotypes (AHap1-AHap6, Table 8). These included 26 deletion sites between positions 393–418 bp, one insertion at position 418 bp, and 10 additional insertion sites spanning 419–428 bp. The remaining three were SNPs at positions 187, 211, and 329 bp.

**Table 6:** Sequence variation within the *trnK(UUU)* gene of *A. eriantha* related to haplotypes.

*trnK(UUU)* Haplotypes	base variation							Genbank accession number
	91	98	109	155	427	435	438	
THap1	A	G	C	A	G	C	C	PX797367
THap2	C	*	*	*	*	*	*	PX874273
THap3	*	*	*	*	A	*	*	PX874274
THap4	*	*	A	G	*	*	A	PX874275
THap5	C	*	*	*	*	G	*	PX874276
THap6	*	A	*	*	*	*	*	PX874277

“*” indicates identity with THap1.

**Table 7:** Sequence variation within the *ycf1* gene of *A. eriantha* related to haplotypes.

*ycf1* Haplotypes	base variation										Genbank accession number
	139	165	171	264	274	275	276	277	278	279	
YHap1	T	C	C	C	A	A	A	G	A	A	PX423730
YHap2	A	*	*	*	*	*	*	*	*	*	PX423731
YHap3	*	T	T	T	*	*	*	*	*	*	PX423732
YHap4	*	*	*	*	–	–	–	–	–	–	PX423733

“*” indicates identity with YHap1; “–” indicates nucleotide deletion.

**Table 8:** Sequence variation within the *atpH_atpI* gene of *A. eriantha* related to haplotypes.

*atpH_atpI* Haplotypes	base variation																																										
	187	211	329	393	394	395	396	397	398	399	400	401	402	403	404	405	406	407	408	409	410	411	412	413	414	415	416	417	418	419	420	421	422	423	424	425	426	427	428	429	430	431	Genbank accession number
AHap1	A	G	T	T	A	A	T	A	T	A	A	T	T	A	A	G	T	A	T	T	A	A	T	A	G	A	A	T	T	A	A	G	T	A	G	A	G	G	T	A	G	C	PX797366
AHap2	T	*	*	*	*	*	*	*	*	*	*	*	*	*	*	*	*	*	*	*	*	*	*	*	*	*	*	*	*	*	*	*	*	*	*	*	*	*	*	*	*	*	PX874278
AHap3	*	A	*	*	*	*	*	*	*	*	*	*	*	*	*	*	*	*	*	*	*	*	*	*	*	*	*	*	*	*	*	*	*	*	*	*	*	*	*	*	*	*	PX874279
AHap4	*	*	*	–	–	–	–	–	–	–	–	–	–	–	–	–	–	–	–	–	–	–	–	–	–	–	–	–	–	*	*	*	*	*	*	*	*	*	*	*	*	*	PX874280
AHap5	*	*	G	*	*	*	*	*	*	*	*	*	*	*	*	*	*	*	*	*	*	*	*	*	*	*	*	*	*	*	*	*	*	*	*	*	*	*	*	*	*	*	PX874281
AHap6	*	*	*	*	*	*	*	*	*	*	*	*	*	*	*	*	*	*	*	*	*	*	*	*	*	*	*	*	A	T	T	A	A	T	A	G	A	A	T	T	A	A	PX874282

“*” indicates identity with AHap1; “–” indicates nucleotide deletion; shaded cells indicate base insertions.

**Fig 5:**
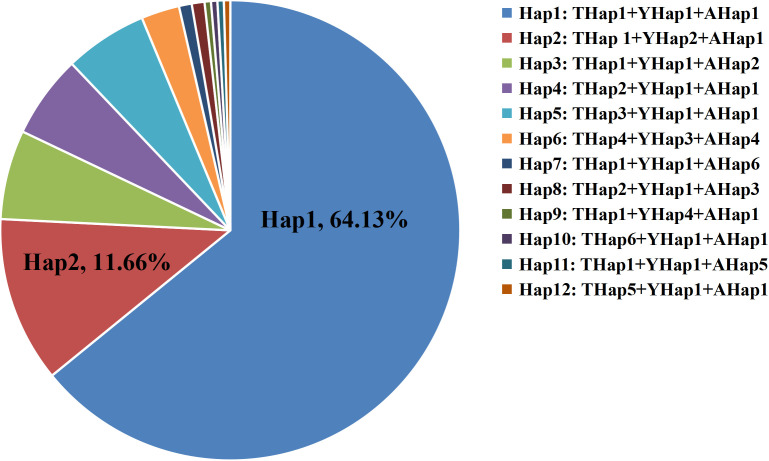
Haplotype proportions derived from combined analysis of three genes (*trnK(UUU)*, *ycf1*, and *atpH_atpI*).

A combined analysis of the three barcode regions (*trnK(UUU)*, *ycf1*, and *atpH_atpI*) identified 12 composite haplotypes (Hap1-Hap12, Fig 5). Among them, Hap1 was the most abundant and widely distributed, present in 64.13% of all samples, followed by Hap2, accounting for 11.66%. Notably, six regions possessed unique haplotypes. For example, Su Baoding in Huaihua City, Hunan Province, was found to harbor two exclusive haplotypes—Hap5 and Hap6. The unique mutation site 427A in *trnK(UUU)* defines Hap5, while Hap6 is characterized by multiple specific variants: *trnK(UUU)* positions 109A, 155G, 438A, *ycf1* positions 165T, 171T, 264T, and a deletion spanning *atpH_atpI* 393–418 bp. These markers enable precise differentiation of Su Baoding germplasm from those of other regions. Other region-specific haplotypes include: Hap8 (unique to Anfu County, Jian City, Jiangxi Province), defined by 91C in *trnK(UUU)* and 211A in *atpH_atpI*; Hap11 (Suichuan County, Ji’an City), characterized by 329G in *atpH_atpI;* Hap12 (Yanshan County, Shangrao City), with specific sites 91C and 435G in *trnK(UUU)*; Hap10 (Jiangkou County, Tongren City, Guizhou Province), defined by 98A in *trnK(UUU)*; and Hap7 (Huangfang Township, Jianning County, Sanming City, Fujian Province), distinguished by a series of insertions at *atpH_atpI* positions 418A, 419T, 420T, 421A, 422A, 423T, 424A, 425G, 426A, 427A, and 428T. Additionally, Hap3 was shared exclusively by samples from Yangjiaping (Dexing City), Yanshan County, and Yushan County in Jiangxi Province, and is characterized by a specific mutation at 187A in *atpH_atpI*. Similarly, Hap9, shared between Anfu County (Jian City) and Laozhushan (Yichun City), was defined by a six-base deletion at positions 274–279 in *ycf1*. These region-specific haplotypes may serve as molecular markers for geographic origin authentication of *A. eriantha*. At the provincial level, Hunan Province had two specific haplotypes (Hap5 and Hap6), Jiangxi Province had five (Hap3, Hap8, Hap9, Hap11, Hap12), and Guizhou and Fujian provinces each had one (Hap10 and Hap7, respectively). The shared haplotypes Hap1 and Hap2 were present across Jiangxi, Guizhou, Fujian, and Zhejiang, while Hap4 was shared among Hunan, Jiangxi, Fujian, and Zhejiang (Table 2).

### Genetic distance analysis of *A. eriantha* haplotypes

Genetic distance analysis was conducted using MEGA X software based on the concatenated sequences of three DNA barcode regions (*trnK(UUU)*, *ycf1*, and *atpH_atpI*). As shown in Table 9, the pairwise genetic distances among the 12 haplotypes ranged from 0 to 1.96%, with an average genetic distance of 0.51%, indicating relatively low genetic divergence among *A. eriantha* samples. The maximum genetic distance (1.96%) was observed between Hap6 and Hap7, suggesting the longest phylogenetic separation. The minimum genetic distance (0.07%) occurred between the following haplotype pairs: Hap1 and Hap2, Hap1 and Hap3, Hap1 and Hap4, Hap1 and Hap5, Hap1 and Hap10, Hap1 and Hap11, Hap4 and Hap8, Hap4 and Hap7, and Hap4 and Hap12, indicating a close genetic relationship. Notably, Hap1 showed a genetic distance of only 0.07% with six other haplotypes, and was also the most widely distributed haplotype across the sampled regions. Based on these findings, we hypothesize that Hap1 represents the ancestral haplotype of *A. eriantha*, from which other derived haplotypes have evolved. In contrast, Hap6 exhibited relatively large genetic distances from all other haplotypes, with values ranging from 1.47% to 1.96% (specifically: 1.47%, 1.54%, 1.54%, 1.54%, 1.54%, 1.96%, 1.61%, 1.89%, 1.54%, 1.54%, and 1.61%), suggesting a more distant phylogenetic relationship and potentially representing an isolated or divergent lineage within the species.

**Table 9:** Genetic distance of haplotypes in joint analysis.

Haplotypes	Hap1	Hap2	Hap3	Hap4	Hap5	Hap6	Hap7	Hap8	Hap9	Hap10	Hap11	Hap12
Hap1												
Hap2	0.07%											
Hap3	0.07%	0.14%										
Hap4	0.07%	0.14%	0.14%									
Hap5	0.07%	0.14%	0.14%	0.14%								
Hap6	1.47%	1.54%	1.54%	1.54%	1.54%							
Hap7	0.49%	0.56%	0.56%	0.56%	0.56%	1.96%						
Hap8	0.14%	0.21%	0.21%	0.07%	0.21%	1.61%	0.63%					
Hap9	0.42%	0.49%	0.49%	0.49%	0.49%	1.89%	0.91%	0.56%				
Hap10	0.07%	0.14%	0.14%	0.14%	0.14%	1.54%	0.56%	0.21%	0.49%			
Hap11	0.07%	0.14%	0.14%	0.14%	0.14%	1.54%	0.56%	0.21%	0.49%	0.14%		
Hap12	0.14%	0.21%	0.21%	0.07%	0.21%	1.61%	0.63%	0.14%	0.56%	0.21%	0.21%	

### Phylogenetic analysis of *A. eriantha* haplotypes

**Fig 6:**
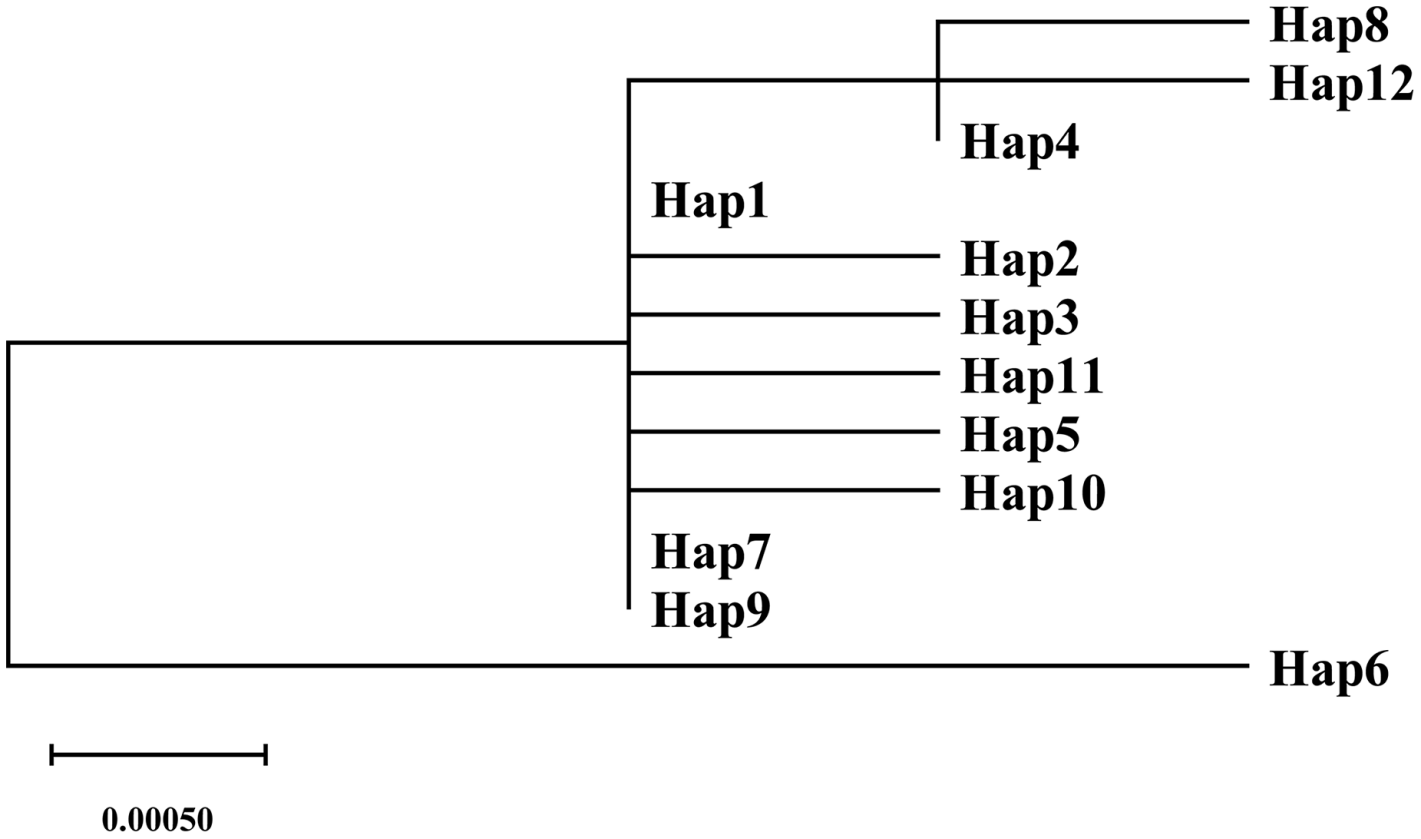
Haplotypes ML tree of combined analysis of three genes (*trnK(UUU)*, *ycf1*, and *atpH_atpI*).

After aligning the haplotype sequences obtained from the joint analysis of three genes, an ML tree was constructed. The 12 haplotypes primarily formed three distinct branches (Fig 6). Hap6 clustered independently into a single branch, exhibiting a relatively distant genetic relationship with the other haplotypes. This finding is consistent with the results of genetic distance analysis, which indicated significant differences in genetic distance and phylogenetic relationships between Hap6 and the other 11 haplotypes.

## Discussion

In this study, chloroplast genome sequences of *A. eriantha* from three different geographic regions in China were analyzed. The results revealed a highly conserved structural organization, strong stability in key functional genes, and similar patterns of variation site distribution across samples. All three chloroplast genomes were circular double-stranded DNA molecules ranging from 156,955–157,100 bp in length, exhibiting the canonical quadripartite structure typical of angiosperm chloroplast genomes. Each genome contained a LSC region, a SSC region, and a pair of IR regions, with a total of 88 annotated genes. These findings are consistent with the report by Tang et al. [12], who sequenced the chloroplast genome of *A. eriantha* from the National Kiwifruit Germplasm Repository at Wuhan Botanical Garden. The genome size and organization observed in this study are in close agreement with previously published data. Across all three *A. eriantha* chloroplast genomes, the gene content and gene order—particularly genes involved in photosynthesis and ribosomal protein synthesis—were found to be highly conserved. Essential functional genes such as *psbA* and *rbcL* (core components of the photosynthetic apparatus), ribosomal RNAs (*rrn* genes), and housekeeping genes such as *accD* and *atpA* showed over 99% sequence identity, indicating strong conservation at the intraspecific level. Comparative genome analysis using mVISTA revealed that coding regions were more conserved than non-coding regions, and that IR regions exhibited greater stability than single-copy regions. This observation is in agreement with patterns reported in other plant species. For instance, chloroplast genomes of Magnoliaceae species are characterized by a typical quadripartite structure, conserved gene arrangement, and minor variation in genome size due to contraction or expansion at the IR/SC boundaries [20]. Similarly, chloroplast genomes of various cultivated pomegranate (*Punica granatum*) varieties exhibit the typical quadripartite architecture of angiosperms, with highly conserved gene content and order and extremely low levels of sequence diversity [21].

DNA barcoding plays a crucial role in the identification of medicinal plant species and the intraspecific classification of germplasm resources, offering advantages such as simplicity, efficiency, and objectivity. In recent years, this technique has also achieved substantial progress in the taxonomic resolution of *Actinidia* species. For example, Ding et al. [22] integrated chloroplast-specific SNP markers with nuclear SCoT markers to analyze the genetic diversity, population structure, and phylogenetic relationships of 55 *Actinidia* samples. Their analysis revealed a total of 15 haplotypes, enabling the precise differentiation of specific *Actinidia* taxa, such as *A. valvata* and *A. arguta*. Similarly, He et al. [15] sequenced and analyzed the chloroplast genome of *Actinidia deliciosa*, identifying four polymorphic chloroplast DNA regions (*atpF-atpH*, *atpH-atpI*, *atpB*, and *accD*) that were proposed as useful molecular markers for further population genetics studies. Based on comparative analysis of the chloroplast genome, this study identified *trnK(UUU)*, *ycf1*, and *atpH_atpI* as potential species-specific DNA barcode regions for *A. eriantha*. A total of 223 samples were collected from 21 locations across six provinces in China. Combined analysis of the three barcode regions identified 12 distinct haplotypes (Hap1-Hap12). Among them, Hap1 was the most common and widely distributed haplotype across all sampled regions. Importantly, six haplotypes were found to be unique to specific geographic regions, suggesting their potential utility as DNA barcode markers for *A. eriantha* germplasm identification.

Based on the combined analysis of the chloroplast genes *trnK(UUU)*, *ycf1*, and *atpH_atpI*, this study successfully achieved intraspecific discrimination in *A. eriantha*. These results support the view proposed by Samarina et al. [23] that, although the chloroplast genome is generally conserved in overall structure, combining specific non-coding regions with highly variable coding genes can still provide sufficient phylogenetic information at the intraspecific level to enable effective differentiation. Samarina et al. highlighted that multiple chloroplast regions, such as *trnE-UUC*/*trnT-GGU*, *psbA_trnH*, *trnL_trnF*, *trnK*, *rpoC1*, *ycf1-a*, *rpl32_trnL*, *trnH_psbA*, and *matK, exhibit* notable performance in intraspecific diversity analysis. They further suggested that selecting and combining three to four of these regions could form an efficient DNA barcoding system applicable for cultivar-level identification. The *ycf1* gene demonstrated high polymorphism in our study, consistent with previous findings. Dong et al. indicated that *ycf1* is one of the most rapidly evolving coding genes in the chloroplast genome of seed plants [24]. In addition, the *trnK(UUU)_matK* locus and the *atpH_atpI* intergenic region also provided critical discriminatory information in this work. Both regions have been previously recognized as highly variable, and Shaw et al. (2007), in a comparative study of chloroplast genomes, explicitly recommended highly variable regions such as *trnK(UUU*), *atpH_atpI*, and *petL_psbE* as effective molecular markers for phylogenetic analysis [25].

It should be noted, however, that chloroplast DNA (cpDNA) in *Actinidia* species is paternally inherited [26,27]. As a uniparental genetic marker, cpDNA may not fully capture the species’ overall genetic variation. The genetic structure observed in this study based on cpDNA was primarily shaped by the spatial distribution of pollen flow rather than seed dispersal. Populations defined by cpDNA haplotypes essentially reflect shared paternal ancestry, rather than maternal origins. This explains why cpDNA patterns may not always align perfectly with geographical distributions and confirms the role of male-mediated gene flow in shaping the genetic diversity of *A. eriantha*. To build on these findings, future research could integrate nuclear genomic markers. Such a combined approach would allow the separate histories of seed and pollen dispersal to be fully reconstructed, thereby providing a more comprehensive understanding of the genetic diversity in *A. eriantha*.

The genetic distance analysis based on haplotypes of the three concatenated genes produced results consistent with the phylogenetic tree. The maximum genetic distance (1.96%) was observed between Hap6 and Hap7, indicating the most distant relationship. This was followed by the distances between Hap6 and Hap9, Hap8, and Hap12, which were 1.89%, 1.61%, and 1.61%, respectively. In the phylogenetic tree, Hap6 formed a distinct clade, demonstrating significant divergence from other haplotypes. Hap6, unique to Su Baoding in Huaihua City, Hunan Province, and Hap7, unique to Huangfang Township, Jianning County, Sanming City, Fujian Province, originated from geographically isolated regions, with Jiangxi Province acting as a physical barrier between them. This long-term geographic isolation may have contributed to their pronounced genetic divergence. In contrast, Hap8, Hap9, and Hap12, all exclusive to Jiangxi Province, were genetically distant from Hap6 due to their occurrence in a different and geographically distant province. Notably, Hap8 (Anfu County, Jian City) and Hap12 (Yanshan County, Shangrao City) clustered into the same phylogenetic clade and shared a very close genetic relationship with a pairwise genetic distance of 0.14%, consistent with their adjacent geographic locations. Research indicates that the distribution patterns of certain haplotypes among different populations of *A. eriantha* are associated with geographical isolation, a phenomenon likely attributable to factors such as local environmental adaptation, historical legacy effects, or dispersal limitations shaping population characteristics. These findings reveal a complex interactive relationship between geographical factors and population structure, though the specific underlying mechanisms require further in-depth investigation. In a study on *Boechera stricta*, Lee et al. [28] employed regression analysis to evaluate the relative contributions of environmental adaptation and geographical isolation to population variation patterns. Their research demonstrated that environmental selection plays a critical role in shaping intraspecific variation across the species’ natural distribution, and that the synergistic effect of geographical and environmental factors leads to a more pronounced and significant population structural response than geographical isolation alone.

In summary, this study analyzed the chloroplast genomes of *A. eriantha* collected from three different regions in China. All three genomes exhibited the typical circular quadripartite structure characteristic of angiosperm chloroplasts. Comparative genomic analysis revealed that the regions *trnK(UUU)*, *ycf1*, and *atpH_atpI* can serve as potential species-specific DNA barcodes for *A. eriantha*. Based on the combined analysis of these three loci, a total of 223 samples from 21 locations across six provinces in China were examined, resulting in the identification of 12 distinct haplotypes (Hap1-Hap12). Notably, six haplotypes were found to be unique to specific geographic regions, and these region-specific haplotypes may serve as effective molecular markers for tracing the geographic origin of *A. eriantha*. Based on genetic distance and phylogenetic analysis, the 12 haplotypes showed limited differences between haplotypes and were primarily resolved into three major clades. Hap6 formed a distinct branch alone, demonstrating relatively larger divergence from other haplotypes. These findings provide important references for determining the geographical origin of *A. eriantha*, as well as for the conservation, utilization of germplasm resources, and breeding programs.
